# Evaluation of Leg Length Difference on Perioperative Radiographs of Total Hip Arthroplasty Considering Lower Limb Malposition

**DOI:** 10.7759/cureus.70790

**Published:** 2024-10-03

**Authors:** Yasuhiko Kokubu, Shinya Kawahara, Yusuke Ayabe, Goro Motomura, Satoshi Hamai, Toshihiko Hara, Yasuharu Nakashima

**Affiliations:** 1 Department of Orthopedic Surgery, Graduate School of Medical Sciences, Kyushu University, Fukuoka, JPN; 2 Department of Orthopedic Surgery, Aso-Iizuka Hospital, Iizuka, JPN

**Keywords:** digitally reconstructed radiography, hip, leg length, malpositioning, total hip arthroplasty

## Abstract

Background: During total hip arthroplasty (THA) in lateral decubitus, perioperative radiography allows the surgeon a simple evaluation of the leg length difference (LLD) by measuring the proximal femoral length. However, the effect of femoral malpositioning on proximal femoral length measurements during the evaluation of perioperative radiographs is not adequately understood. We aimed to (1) investigate the effects of malposition on proximal femoral length using three-dimensional computer simulations and (2) verify whether a simple correction formula can improve the accuracy of LLD evaluation on perioperative radiographs.

Methods: We analyzed 86 patients who underwent THA. Digitally reconstructed radiography (DRR) images were reconstructed in various limb positions (femoral abduction, adduction, and flexion), and proximal femoral length measurements in those malpositions were simulated. Additional morphological measurements of the femoral neck torsion angle in the sagittal plane were performed to elucidate the simulation findings. The malposition angle of abduction-adduction was evaluated with actual perioperative radiographs, and trigonometric correction was attempted.

Results: The leg length measurement decreased as the femoral DRR image shifted from neutral to abduction and adduction, demonstrating approximately 1 mm per 10° of abduction or adduction. The leg length measurement increased as the femoral image shifted from neutral to 10° and 20° of flexion, demonstrating approximately 3 mm per 10° of flexion. With a peak at 20° of flexion, the proximal femoral length measurement decreased in the DRR images at 30°, 40°, 50° and 60° of flexion. The femoral neck torsion angle was 21.1 ± 5.6° on the operative side. The effect of coronal malposition on leg length discrepancy was so small that the difference following trigonometric correction was not statistically significant (p=0.108).

Conclusion: In the present simulation, coronal malposition had a small effect on LLD evaluation. As the femoral neck has a torsion of approximately 20°, the proximal femoral length is projected the longest when the femur is flexed 20°. With careful positioning of the limb in the coronal plane, the use of a correction formula for LLD evaluation would not be necessary. Surgeons should ensure that both lower limbs are in the same position in the sagittal plane during THA in lateral decubitus.

## Introduction

Appropriate positioning of the acetabular and femoral components was reported to be important in determining the dislocation risk, acetabular polyethylene wear, and need for revision surgery [1,2] during total hip arthroplasty (THA). Furthermore, postoperative leg length difference (LLD) causes limping, low back pain, and uneven weight distribution, which decreases patient satisfaction [3,4]. Computer-assisted orthopedic surgery (CAOS) such as surgical navigation systems or robotic-arm-assisted systems are useful for evaluating both the position of each component and leg length during surgery [5-7]; however, these devices require additional capital expenditures and cannot be used in every institution, especially small and medium volume institutions. Conversely, perioperative radiography can be performed in some institutions without additional instrumentation and capital expenditures [8,9], as it has been reported to improve acetabular and femoral component placement as a supportive tool [4].

For the simple evaluation of LLD on perioperative radiographs, the surgeon alternatively uses bony landmarks, such as the most prominent part of the lesser trochanter and the trans-teardrop line, to measure the proximal femoral length [5,10]. For accurate assessment, the position of the pelvis and lower limbs should be carefully set to ensure a neutral position in each plane. However, it is not always easy to place the bilateral femurs in the same position - especially during THA in the lateral decubitus position (posterolateral approach, Rottinger approach, and Hardinge approach) [11] - and LLD may not be accurately evaluated due to measurement errors caused by asymmetrical leg position (such as abduction, adduction, and flexion). Additionally, the effect of malposition on LLD measurements has not been adequately understood. Theoretically, the effect of coronal malposition (abduction and adduction) on proximal femoral length measurements can be adjusted with mathematical formulae; however, its clinical use has not been clarified.

The objectives of this study were to (1) investigate the effects of malposition on LLD using three-dimensional (3D) computer simulations, and (2) verify whether a simple correction formula can improve the accuracy of the LLD evaluation on perioperative radiographs. We hypothesized that proximal femoral length is measured to be shorter when the lower limb is adducted, abducted, or flexed. We also hypothesized that a simple correction formula can improve the accuracy of the LLD evaluation on perioperative radiographs.

## Materials and methods

Patients

**Figure 1:**
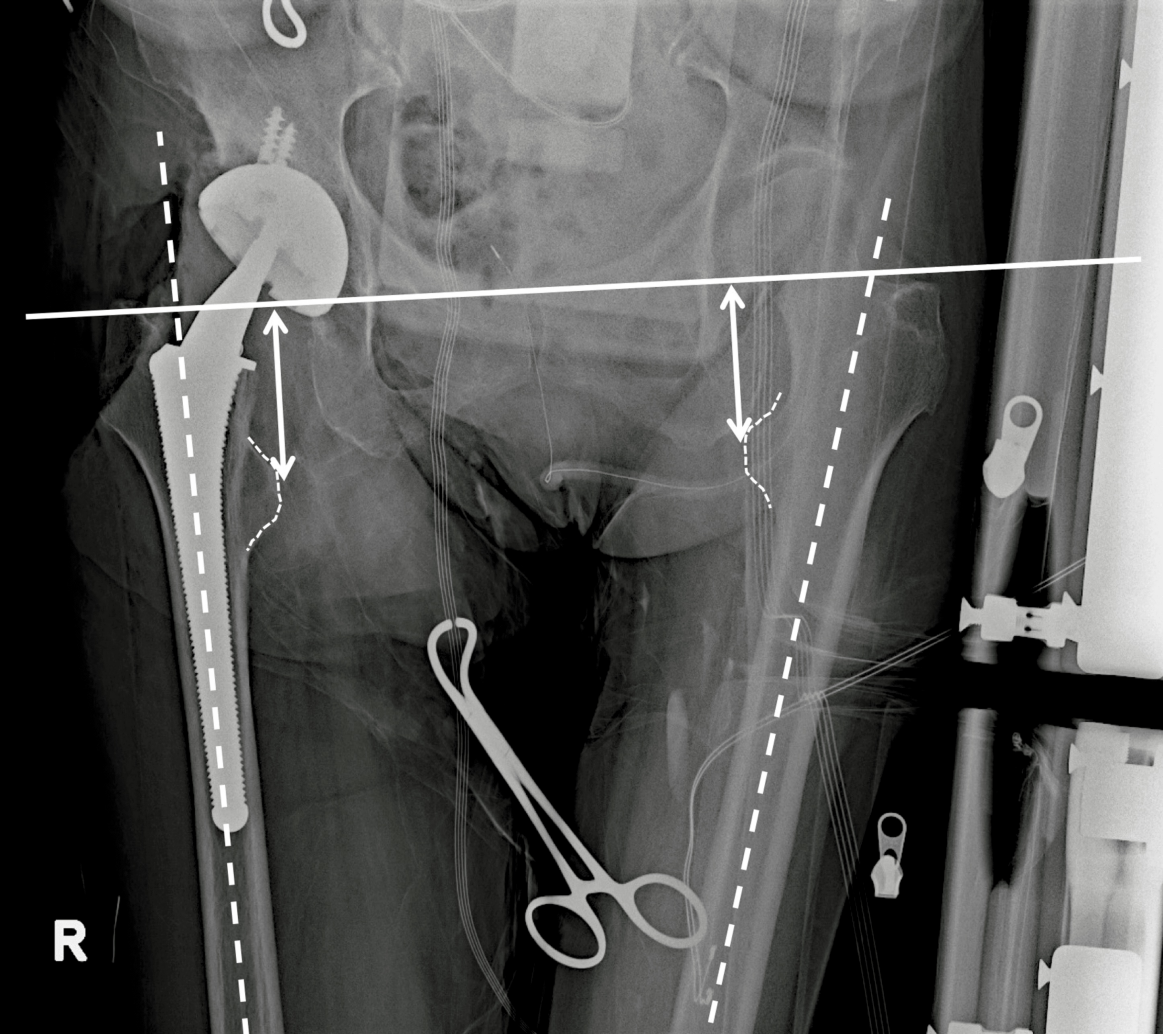
Perioperative radiograph Measurement of leg length on a perioperative radiograph. The vertical distance (arrows) is measured from the trans-teardrop line (the solid line) to the most prominent point of the lesser trochanter. The broken line indicates the femoral anatomical axis.

**Figure 2:**
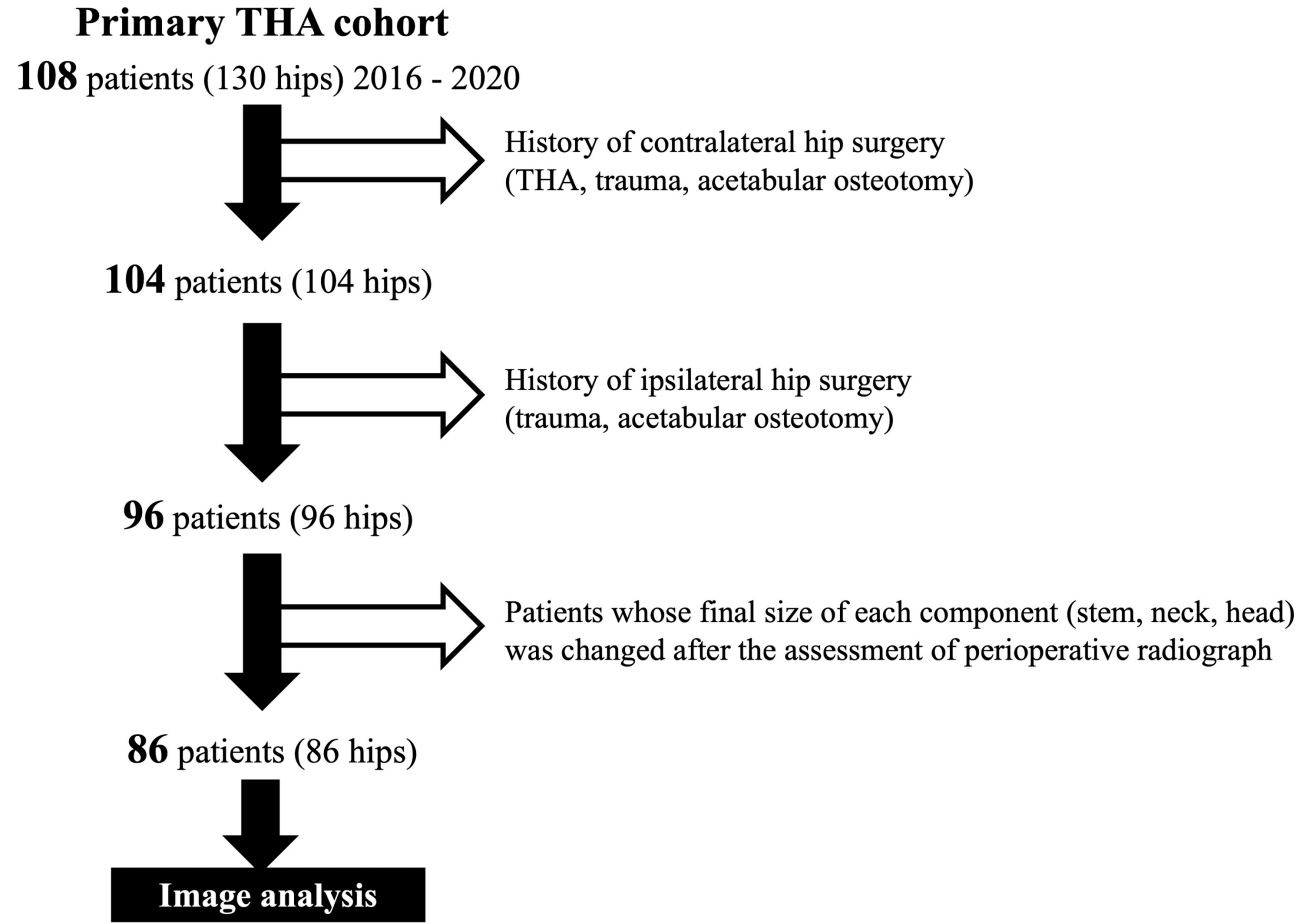
A flowchart detailing patient selection THA - total hip arthroplasty

The study group comprised 108 consecutive patients (130 hips) who underwent THA between September 2016 and March 2020 using one specific femoral cementless component (PerFix910 stem; Kyocera, Osaka, Japan). Patients with osteoarthritis (primary and secondary to dysplasia), osteonecrosis of the femoral head, or subchondral insufficiency fracture of the femoral head were included, while patients with a history of hip or pelvic surgery (osteotomy and trauma) were excluded. All THAs were performed through a posterolateral approach in the lateral decubitus position. The pelvis was fixed with bilateral anterior superior iliac spines and the sacrum. The thorax was also fixed with sternal support in the front and retro-thoracic support in the back. Preoperative and perioperative pelvic radiographs were routinely taken in our institution using a portable radiograph device (Figure 1). The irradiation origin was centered at the superior margin of the pubic symphysis, and the film was held vertically and positioned at the back of the pelvis, parallel to the trunk. The distance between the irradiation origin and film was consistently maintained (180 cm). Preoperative radiographs were taken just after setting the lateral position, and the pelvic position was carefully adjusted to match the target position by tilting or rotating the operating table. Perioperative radiographs were taken after the trial reductions using the permanent acetabular component and the trial femoral broach, head, neck, and liner. The leg on the operative side was placed on a Mayo stand so that it was parallel to the ground. If the surgeon judged that the limb malposition was severe during the radiography, the quality of the images was considered inadequate, and the radiographs were re-taken. Patients whose final size of each component (stem, neck, head) was changed after the assessment of perioperative radiographs were excluded from the study. After the eligibility assessment, 86 patients (86 hips) were included in this study (Table 1, Figure 2). All patients were Japanese and provided informed consent before participation. The study procedures were approved by the local institutional review board (No.30-91) and conducted in accordance with the 1964 Declaration of Helsinki.

**Table 1:** Patient details Note: Values are given as the mean and standard deviation. OA, Osteoarthritis; Osteonecrosis of the femoral head, ON; SIF, Subchondral insufficiency fracture of the femoral head

Variable	Data (n=86)
Age (years)	67.3 ± 10.0
Sex, male (n)	6 (7%)
Sex, female (n)	80 (93%)
Side, right (n)	52 (60%)
Side, left (n)	34 (40%)
Perioperative diagnosis, OA (n)	81 (94%)
Perioperative diagnosis, ON (n)	3 (4%)
Perioperative diagnosis, SIF (n)	2 (2%)

Methods of simulation

3D Coordinate System Definition and DRR Image Reconstruction From Postoperative Computed Tomography

**Figure 3:**
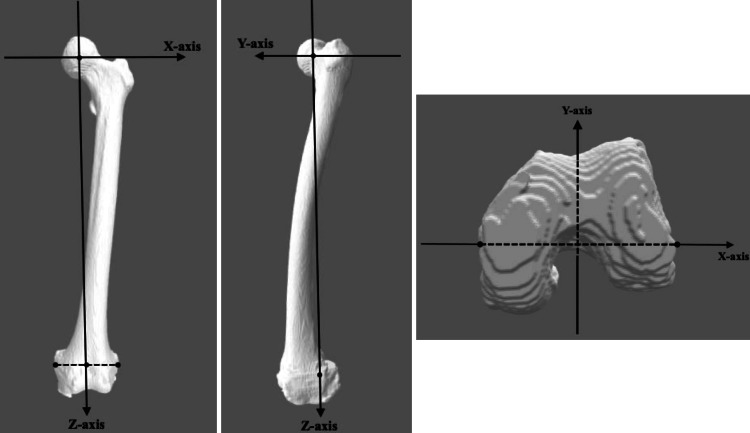
The femoral coordinate system.

Computed tomography (CT) (Revolution CT; GE Healthcare Japan, Tokyo, Japan) was routinely performed one week postoperatively in all cases at levels ranging from the whole pelvis to the knee joint at 1.25 mm intervals (1.25 mm thickness with a field of view of 400 and pitch of 1.375). CT images were acquired as Digital Imaging and Communications in Medicine format (DICOM) data from the CT system server. The DICOM datasets were then imported into a 3D planning software (3D template; Kyocera, Osaka, Japan), and 3D femoral bone models were reconstructed and segmented in the software. In order to simulate hip flexion and adduction-abduction around the femoral head, the International Society of Biomechanics (ISB) coordinate system was used to represent the femur [12]. The femoral mechanical axis was defined as the line connecting the femoral head center and the midpoint of the transepicondylar axis (connecting the medial and lateral epicondyles of the distal femur) [12,13], and the femoral head center and femoral mechanical axis was defined as the origin and longitudinal axis (z-axis), respectively (Figure 3). The digitally reconstructed radiography (DRR) coronal image based on the 3D femoral coordinate system described above was defined as the neutral femoral radiograph. 

Simulation of the Femoral Position

**Figure 4:**
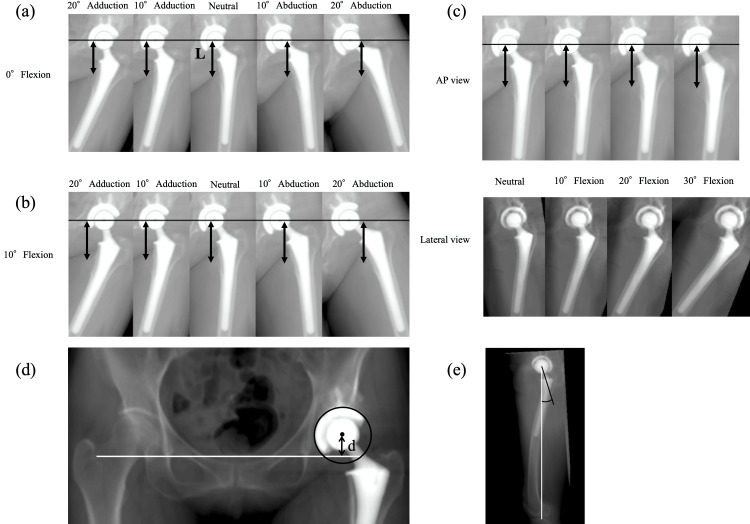
Image analysis (a-c) Digitally reconstructed radiography images were reconstructed in various limb positions. The vertical distance from the X-axis (the solid black line) to the most prominent point of the lesser trochanter (the black arrow, L) was measured on each image. (d) The vertical distance (the black arrow, d) from the epiphyseal head center (the solid black circle) to the trans-teardrop line (the solid white line) was used for leg length measurement. (e) On the digitally reconstructed sagittal image, the torsion angle was measured of the femoral neck axis (the solid black line) relative to the femoral mechanical axis (the white solid line).

The DRR images were reconstructed in various limb positions by abduction, adduction, and flexion of the mechanical axis. First, DRR images at 10° and 20° of abduction or adduction relative to the neutral radiograph were reconstructed (Figure 4a). Second, DRR images were reconstructed with 10° and 20° of femoral flexion relative to the neutral radiograph; DRR images at 10° and 20° of abduction or adduction with 10° and 20° of flexion were then reconstructed (Figure 4b). Third, DRR images were reconstructed at 30°, 40°, 50° and 60° of flexion relative to the neutral radiograph (Figure 4c). The vertical distance in these various DRR images was measured from the x-axis to the most prominent point of the lesser trochanter (“L”; Figure 4a). These simulations were performed on both the operative and nonoperative sides. Next, 3D pelvic bone models were reconstructed and segmented in the software; the pelvic 3D coordinate system was defined based on the anterior pelvic plane [14,15]. Then, the pelvis was realigned in the sagittal plane to simulate pelvic inclination in the supine position, defined as the functional pelvic plane [16,17]. This reference plane was used for the simulation because, in actual surgery, the pelvic position is adjusted according to the preoperative supine radiographs after positioning the patient in the lateral decubitus position. The pelvic DRR image parallel to the functional pelvic plane was reconstructed, and the distance was measured from the femoral head center to the trans-teardrop line (“d”; Figure 4d) [10,18]. Proximal femoral length was defined as the length “L” minus the length “d” for each DRR image. Moreover, additional morphological measurements were performed to validate the simulation results. On the DRR sagittal plane, the torsion angle was measured of the femoral neck axis relative to the femoral mechanical axis (Figure 4e).

LLD Evaluation on the Perioperative Radiograph 

On perioperative radiographs, surgeons generally evaluate the position and alignment of each component (such as the lateral inclination and anteversion of the acetabular component, and the varus-valgus alignment of the femoral broach), LLD, and lateral offset (such as the femoral offset and the global offset). In this study, the bilateral femoral lengths and limb position were focused on and analyzed with OP-A software (Fujifilm, Tokyo, Japan). The vertical distance was measured between the trans-teardrop line and the most prominent point of the lesser trochanter (Figure 1), and the difference between the operative and nonoperative sides was defined as the LLD. Accurate calibration of the image magnification was made by using the acetabular component as a marker with known dimensions, allowing for precise measurements.

Validation of correction formula

**Figure 5:**
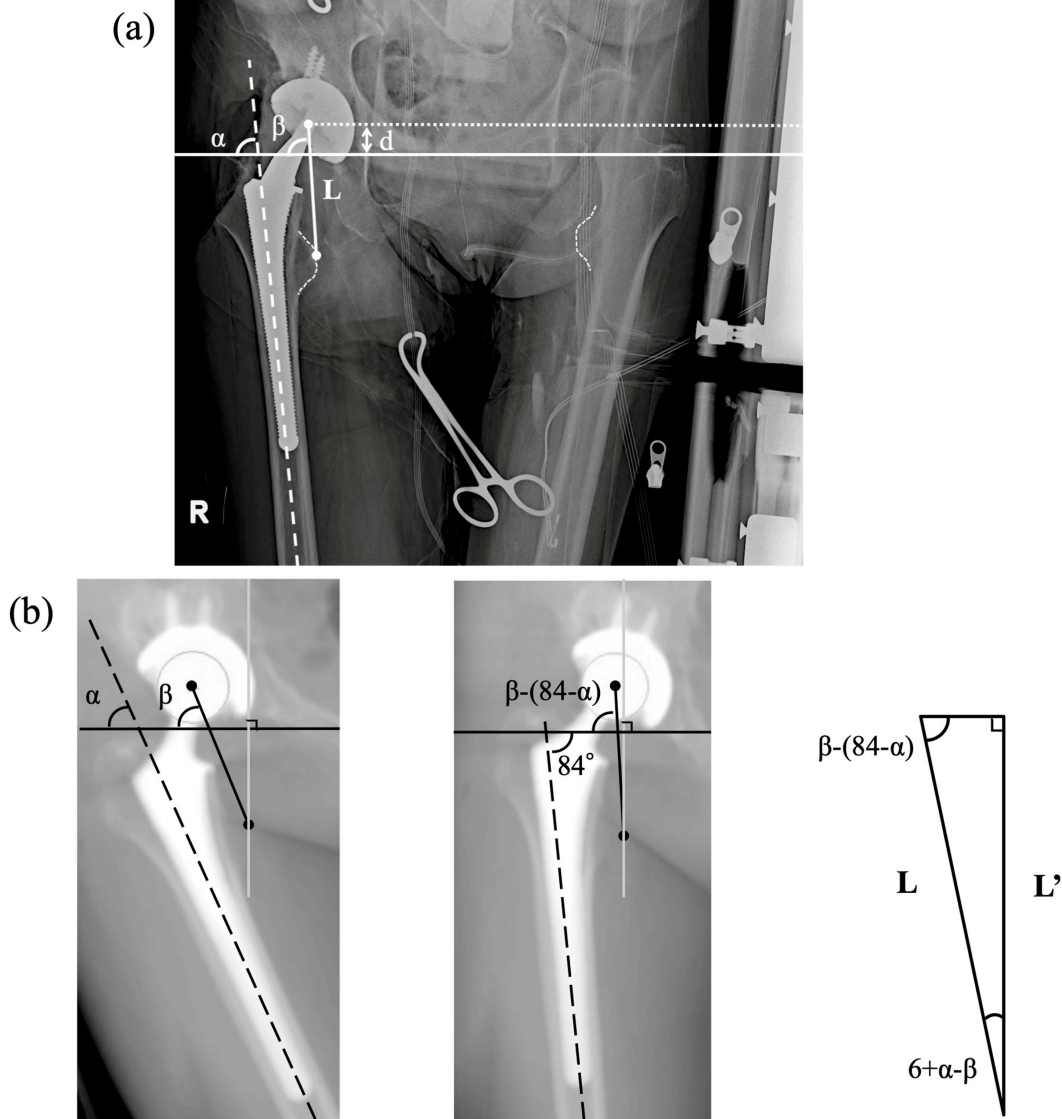
A mathematical correction of the femoral malposition based on the trigonometry (a) Measurement of the distance from the femoral head center to the most prominent point of the lesser trochanter (Line L) and the vertical distance (d) from the femoral head center to the trans-teardrop line (the solid white line). Angles were measured between the trans-teardrop line and the femoral anatomical axis (angle α) and between the trans-teardrop line and line L (angle β). The broken white line indicates the femoral anatomical axis. (b) Line L is abducted by (84–α)° to correct the adduction (left figure) to neutral (center figure). Then, the orthogonal element of line L to the trans-teardrop line (line L') is calculated as follows: L' = L cos (6+α–β). The valgus angle between the femoral anatomical and mechanical axes was set at 6°. The solid black line indicates the trans-teardrop line, while the broken black line indicates the femoral anatomical axis. The solid gray line is orthogonal to the trans-teardrop line and parallel to the femoral functional axis.

The hip joint exhibits adduction/abduction around the femoral head, as demonstrated in previous biomechanical studies [19,20], and simulation of the hip joint motion was conducted under the same simple definition [21,22]. Therefore, on perioperative radiographs, a simple correction of proximal femoral length, influenced by the abduction-adduction position of the bilateral limbs in the coronal plane, was attempted (Figure 5a). A line was drawn from the femoral head center to the most prominent point of the lesser trochanter (line L). Angles were measured between the trans-teardrop line and the femoral anatomical axis (angle α) and between the trans-teardrop line and line L (angle β). Assuming that the trans-teardrop line and femoral mechanical axis are orthogonal, a mathematical correction was made based on the trigonometry (Figure 5b). The valgus angle between the femoral anatomical and mechanical axes was set at 6° [23,24]. Considering the vertical distance from the femoral head center to the trans-teardrop line (d), the corrected proximal femoral length was calculated based on the formula: Corrected proximal femoral length: L’ - d = L cos (6+α-β)° - d.

The “true proximal femoral length” was defined as the leg length in the neutral DRR image reconstructed from postoperative CT scans (described above in detail). Variations of the “true proximal femoral length” were compared before and after mathematical corrections, and variation was assessed as an absolute value. LLDs were also compared before and after correction, and corrections were performed on both the operative and nonoperative sides.

Statistical analysis

On perioperative radiographs, femoral coronal alignment was compared between the operative and nonoperative sides using a t-test. To verify the effects of correction, differences in the “true leg length” were compared before and after correction using a paired t-test. Data analyses of the leg length measurement for each DRR image in different femoral positions were conducted using repeated-measures analysis of variance. Continuous variables were presented as mean ± standard deviation. Statistical analyses were performed using JMP software version 15.0 (SAS Institute, Cary, NC, USA), and statistical significance was set at p<0.05. Post-hoc power analysis was performed using G*Power version 3.1 (Heinrich-Heine-Universität, Düsseldorf, Germany). For a total sample size of 86 and type-I error (α) of 0.05, the study was expected to provide a power (1 - β) of 0.96, 0.99, and 0.99 for detecting an effect size of 0.4, 0.5, and 0.6, respectively. To evaluate intraobserver and interobserver reproducibility, measurements were performed twice by one examiner (YK), and once by another examiner (YA). The intraclass and interclass correlation coefficients were good for all measurements (0.81 to 0.87 and 0.78 to 0.85, respectively).

## Results

Effects of malposition on LLD measurements

**Figure 6:**
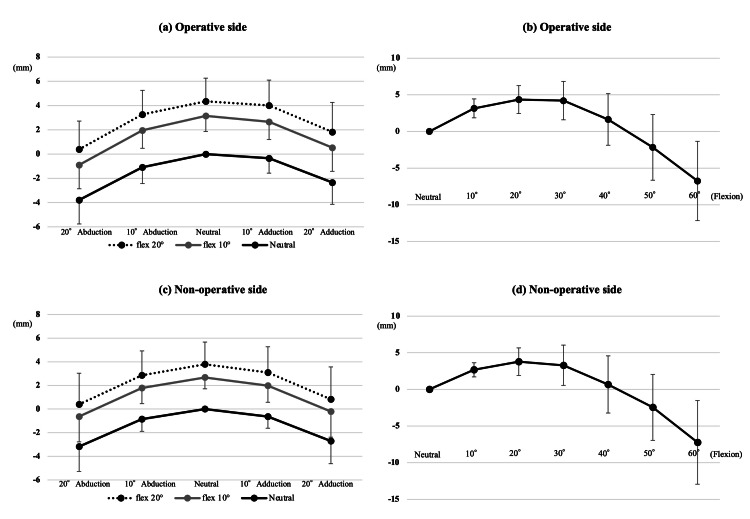
(a-d) Proximal femoral length in various limb positions Line graphs show proximal femoral lengths in various limb positions. Length in neutral (adduction and abduction: 0°; flexion: 0°) was used as the reference value. Black dots indicate mean values, while error bars indicate standard deviations.

**Figure 7:**
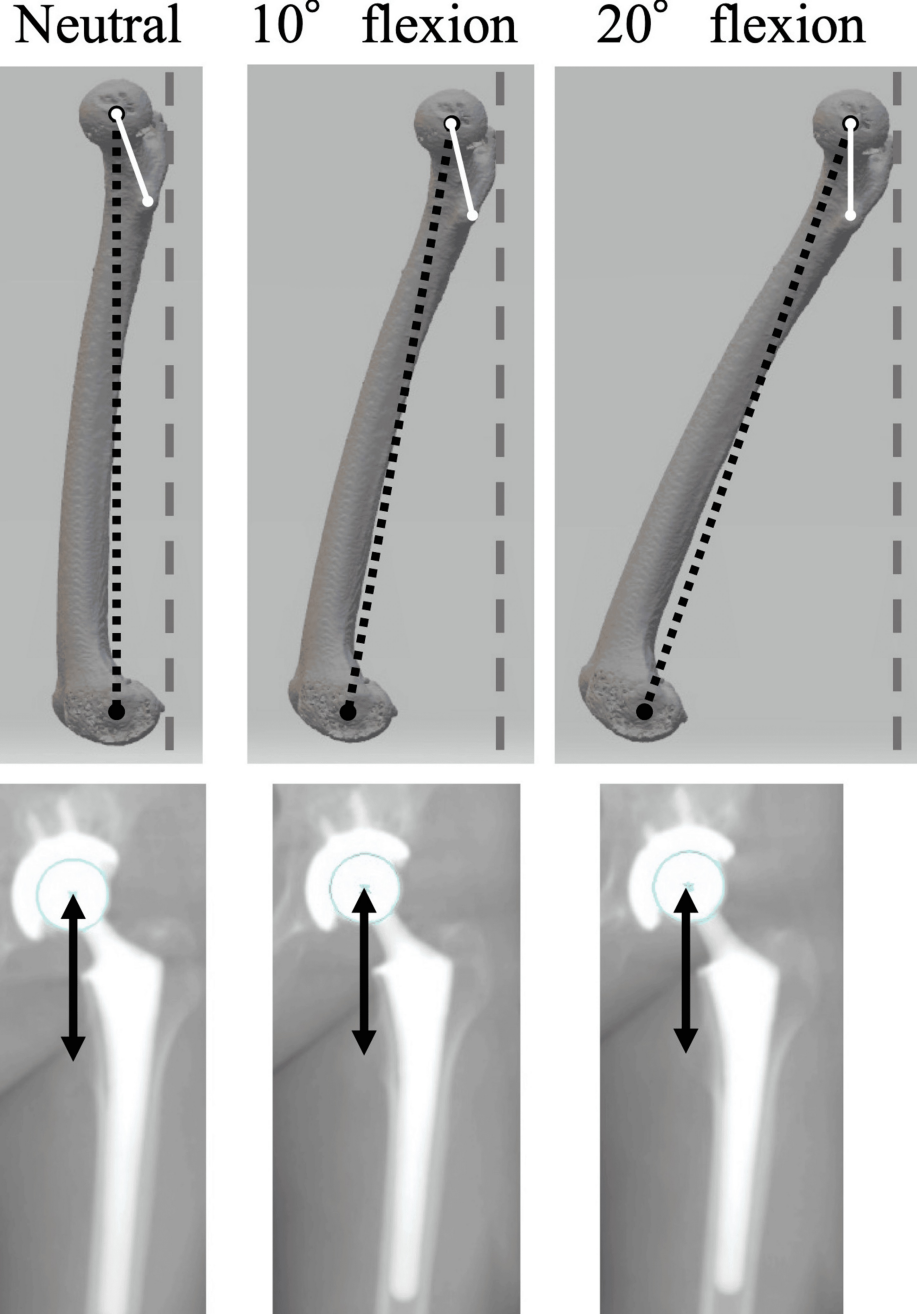
Hip flexion and projected proximal femoral length The solid white line shows the line that connects the femoral head center to the most prominent point of the lesser trochanter. This shows anteversion in comparison to the coronal plane (the broken gray line). With hip flexion, the projected length (the black arrow) is the longest when the line is parallel to the coronal plane.

**Figure 8:**
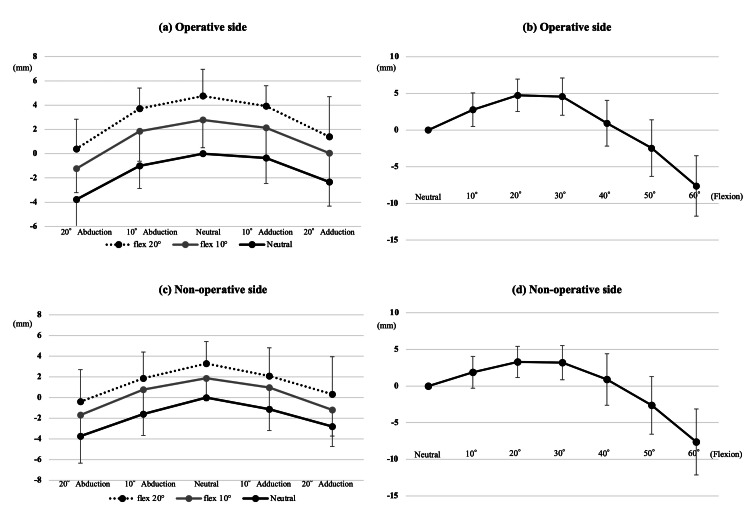
Proximal femoral lengths in various limb positions using another bony landmark Line graphs show proximal femoral lengths in various limb positions. Measurements were obtained using different bony landmarks compared with those in Figure 6. Length in neutral (adduction and abduction: 0°; flexion: 0°) was used as the reference value. Black dots indicate mean values, while error bars indicate standard deviations.

Figures 6a-6d show the simulation results for each malposition. The proximal femoral length significantly decreased as the DRR image shifted from neutral to 10° and 20° of abduction and adduction (Figures 6a, 6c). The proximal femoral length significantly increased on the DRR image with 10° and 20° of flexion relative to the neutral (Figures 6b, 6d) and significantly decreased with abduction and adduction (Figures 6a, 6c). Each 10° of femoral abduction or adduction decreased the proximal femoral length measurements by 1 mm, whereas each 10° of femoral flexion increased measurements by 3 mm. Figures 6b, 6d show the transitions of leg length measurement with flexion. The proximal femoral length significantly increased as the DRR image shifted from neutral to 10° and 20° of flexion (Figure 7); however, with a peak at 20° of flexion, the proximal femoral length significantly decreased on the DRR images at 30°, 40°, 50° and 60° of flexion. There was a significant numerical difference (p<0.001) in proximal femoral length between 20° and 30° of flexion; however, the difference was clinically slight (<0.6 mm on average). Proximal femoral length measurement showed a similar trend with malposition on both the operative and nonoperative sides. On the DRR sagittal image, the torsion angle of the femoral neck axis relative to the femoral mechanical axis was approximately 20°; 21.1 ± 5.6°on the operative side and 18.5 ± 4.5° on the nonoperative side. Regarding the proximal femoral length measured on DRR images, the intraclass correlation coefficients were 0.83 and 0.81 for the operative and nonoperative sides, respectively. Meanwhile, the interclass correlation coefficients were 0.81 and 0.78 for the operative and nonoperative sides, respectively. Regarding femoral neck torsion angle, the intraclass correlation coefficients were 0.88 and 0.86 for the operative and nonoperative sides, respectively, whereas the interclass correlation coefficients were 0.86 and 0.84 for the operative and nonoperative sides, respectively. Considering that proximal femoral length measurements may depend on the definition of anatomical landmarks, additional simulations using different landmarks (measuring the distance from the sciatic tubercle to the inferior margin of the lesser trochanter) are shown in Figure 8, but the trends in measurements were similar.

Validation of correction formula

On the perioperative radiographs, the femoral anatomical axis was 9.8 ± 6.6°of adduction on the operative side and 14.9 ± 5.4° of adduction on the non-operative side. Considering that the angle between the mechanical and anatomical axes was 6°, the femoral mechanical axis was 3.8 ± 6.6° of adduction on the operative side and 8.9 ± 5.4° of adduction on the non-operative side. The adduction angle was significantly larger on the nonoperative side (p<0.001). The results of the correction for femoral malposition are shown in Tables 2, 3. The radiographic measurements of the proximal femoral length relative to the neutral DRR measurements data did not significantly differ before and after correction on either the operative (before correction: 2.7 ± 2.3 mm, after correction: 2.7 ± 2.2 mm, p=0.343) or nonoperative side (before correction: 3.1 ± 2.9 mm, after correction: 3.2 ± 2.9 mm, p=0.305). While the correction slightly reduced the leg length discrepancy between the operative and nonoperative sides (before correction: 3.3 ± 3.1 mm, after correction: 3.1 ± 3.0 mm), it was not statistically significant (p=0.108). Regarding the proximal femoral length measured on the perioperative radiograph, the intraclass correlation coefficients were 0.87 for the operative side and 0.85 for the nonoperative side, whereas the interclass correlation coefficients were 0.85 for the operative side and 0.83 for the nonoperative side.

**Table 2:** Measured distance from the trans-teardrop line to the lesser trochanter on the digitally reconstructed radiography with “neutral” positioning before and after correction on the actual perioperative radiograph Note: Values are given as the mean and standard deviation. DRR, digitally reconstructed radiography

	DRR	Before correction	After correction
Operative side (range, mm)	37.7±6.6 (21.6 to 53.2)	38.9±6.1 (24.3 to 51.1)	39.0±6.1 (24.7 to 49.7)
Nonoperative side (range, mm)	33.5±6.3 (16.7 to 47.8)	34.3±6.7 (14.5 to 50)	34.8±6.8 (14.0 to 49.8)
Leg length difference (range, mm)	4.2±5.1 (-12.8 to 18.4)	4.6±5.8 (-8.6 to 25.2)	4.2±5.9 (-7.5 to 25.3)

**Table 3:** Comparison of the measurement on the perioperative radiograph before and after correction Indicates the absolute value obtained by subtracting the CT measurement from the perioperative radiograph measurement. Note: Values are given as the mean and standard deviation. DRR, digitally reconstructed radiography

	Perioperative radiograph - DRR		
	Before correction	After correction	P-value
Operative side (range, mm)	2.7±2.3 (0.1 to 10.4)	2.7±2.2 (0.1 to 10.4)	0.343
Nonoperative side (range, mm)	3.1±2.9 (0 to 16.8)	3.2±2.9 (0.1 to 17.7)	0.305
Leg length difference (range, mm)	3.3±3.1 (0 to 16.1)	3.1±3.0 (0 to 16.8)	0.108

## Discussion

Main results

Although CAOS has become in wide use in recent years, perioperative radiography is still a useful tool to support a simple assessment of component placement, despite its disadvantages (septic risk, increased operating time, and radiation exposure) [8,25]. The present study investigated the effect of femoral malposition on radiographic LLD measurements. Several studies have shown that patient positioning is critical for accurate component placement during THA in the lateral position [11,14]; however, few have described the effect of patient positioning on intraoperative radiographic evaluations. Debbi et al. [26] performed computer simulations using an anatomical model to demonstrate the possibility of measurement variability caused by femoral malposition. Still, their simulation did not use actual patient data, and the measurement variation was not simulated and quantified in detail. The present study is, therefore, the first to simulate and quantify these variations in detail. 

Effects of malposition on LLD measurements

From our simulation results using various patterns of DRR images, the proximal femoral length measurement decreased as the femoral DRR image shifted from neutral to abduction and adduction, demonstrating approximately 1 mm per 10° of abduction or adduction. Given that actual perioperative radiographs were taken with about 10° of adduction, the measurement error due to malposition in the coronal plane was as small as 1 mm if careful attention was paid to the limb position. Contrary to our hypotheses, the measurements increased with mild hip flexion (0-20°) and then decreased as flexion increased beyond 20°. No previous reports have adequately explained this tendency; however, the proximal femoral morphology may explain this mechanism (Figure 7). The proximal femoral length is the projected length between the bony landmarks of the proximal femur. Therefore, as the simulation indicates, the length of the projected femoral neck is critical. The femoral neck has a torsion of approximately 20° in the sagittal plane; thus, when the femur is flexed 20°, the proximal femur is parallel to the x-ray film in the sagittal plane, and the proximal femoral length is projected the longest. While 10° of adduction or abduction can result in a measurement error of about 1 mm, 10° of flexion can result in an error of about 3 mm, which is relatively large. Although a simplified assessment using a perioperative radiograph differs from the actual measurement of the entire length of the leg, it is necessary to take into account “how the proximal femur is projected” owing to the limb position.

Validation of correction formula

In clinical practice, malpositioning in the coronal plane is difficult to confirm visually during THA in the lateral decubitus position. On average, the femur aligned with 3.8° of adduction on the operative side and 8.9° of adduction on the nonoperative side in the actual perioperative radiographs; adduction was thus larger on the nonoperative side. In many cases, the pelvis on the operative side would tilt distally relative to the nonoperative side on the operating table [14,27], which may explain the larger hip adduction on the nonoperative side. Theoretically, while the effects of the abduction-adduction position of bilateral limbs on radiographic proximal femoral length evaluations would be correctable by a mathematical formula using trigonometry, those of the extension-flexion would not be correctable. However, the change in proximal femoral length measurements under the correction formula was so small - about 0.2 mm on average - and was not statistically significant. This could be explained by the fact that the actual perioperative radiographs contained negligible malposition under careful positioning control. In the present simulation, the finding that coronal malposition had a relatively small effect on proximal femoral length measurements also supported the inapplicability of the correction formula. With careful positioning of the limb in the coronal plane, it is clinically important that the use of a correction formula for LLD evaluation would not be necessary. On the other hand, the measurement error caused by malposition in the sagittal plane was relatively large. In the lateral decubitus position, limb malposition in the sagittal plane can be aptly confirmed visually. It is necessary to pay close attention to the same limb position in the sagittal plane when THA is performed in the lateral decubitus position.

Study limitations 

This study has several limitations; first, the present study did not consider pelvic malposition. The pelvis might be displaced owing to intraoperative maneuvers. Both the thorax and pelvis were fixed but neither was fixed rigidly. In practical surgery, malposition on the pelvic side should also be evaluated. Nevertheless, several methods have been reported to quantify the details of pelvic malposition [14], and obtaining an accurate radiograph of the pelvis has become less difficult. Second, perioperative radiography was performed after the insertion of a trial femoral broach, whereas postoperative CT was performed after the insertion of a permanent femoral component. These components may not be placed in exactly the same alignment. To minimize these mismatches, we included patients with a collared stem due to its high reproducibility of insertion depth and visibility of component rotation. Third, the evaluation may depend on the definition of anatomical landmarks [28,29]. Additional simulations were performed by measuring the distance from the trans-ischial line to the inferior margin of the lesser trochanter, assuming a different landmark from the present study, but the trends in the measurements were similar (Figure 8). In addition, although the intraclass and interclass correlation coefficients were good for all measurements in this study, these variations may also potentially affect the evaluation. Forth, the malposition of the sagittal plane during the perioperative radiography could not be quantified on the images. Fifth, perioperative radiographs during THA are not routinely performed at all facilities. Although CAOS is becoming widely used, it is still a useful option for improving accuracy due to its low cost and convenience.

## Conclusions

The proximal femoral length measured on perioperative radiographs was found to be smaller when the femur was in adduction and abduction, and larger in mild flexion. The effect of coronal malposition (abduction and adduction) on proximal femoral length measurements was so small that mathematical correction of the limb position was unnecessary with careful positioning during radiography. However, the effect of sagittal malposition (flexion) was relatively large, and care should be taken to keep both lower limbs in the same position in the sagittal plane during THA in lateral decubitus.
